# Effects of Methylglyoxal on Intestinal Cells: Insights on Epigenetic Regulatory Enzymes

**DOI:** 10.1002/iub.70067

**Published:** 2025-12-08

**Authors:** Camilla Morresi, Giulia Feliziani, Luisa Bellachioma, Christian Giommi, Rosita Gabbianelli, Laura Bordoni, Gianna Ferretti, Tiziana Bacchetti, Elisabetta Damiani

**Affiliations:** ^1^ Department of Life and Environmental Sciences Polytechnic University of Marche Ancona Italy; ^2^ School of Advanced Studies University of Camerino Camerino MC Italy; ^3^ Unit of Molecular Biology and Nutrigenomics University of Camerino Camerino MC Italy; ^4^ Department of Clinical Experimental Science and Odontostomatology Polytechnic University of Marche Ancona Italy

**Keywords:** epigenetics, inflammatory bowel diseases, methylglyoxal, oxidative stress, ultra‐processed foods

## Abstract

Methylglyoxal (MGO) is endogenously produced under physiological conditions as a by‐product of glycolysis and by autooxidation of glucose and lipid peroxidation. The digestive system can also take up MGO from exogenous sources, especially from ultra‐processed foods. MGO is a highly reactive molecule, able to react with macromolecules forming covalent adducts resulting in advanced glycation end‐products formation. MGO can also enter the nucleus and react with nucleic acids with the formation of MGO‐nucleic acid adducts. The intestinal epithelium is continuously exposed to dietary and endogenous stimuli, including MGO, but the potential harmful role of MGO at the intestinal level has been poorly investigated. Therefore, the aim of the study was to further investigate the effects of MGO in intestinal cells and the molecular mechanisms involved, with particular attention to epigenetic regulatory enzymes such as histone deacetylases (HDAC), ten‐eleven translocation (TET) family enzymes, and DNA methyltransferases (DNMT). Our results demonstrate that MGO exposure induces alterations in intestinal barrier function in differentiated Caco‐2 cells monolayers. Moreover, MGO treatment induces cell apoptosis associated with an increase in cytosolic and mitochondrial reactive oxygen species. MGO‐induced oxidative stress was associated with activation of the NFκB pathway and increased levels of proinflammatory molecules such as TNF‐α and antioxidant enzymes (superoxide dismutase 1 [SOD1] and catalase). The increased expression of γH2AX suggests damage to DNA in MGO‐treated cells. A decrease in HDAC1/2 expression, consistent with the increase in acetylated histone H4 levels, and an inhibition of the expression of TET (TET1, TET2) proteins was observed in MGO‐treated cells. These results suggest that MGO may also disrupt epigenetic homeostasis mechanisms, offering further insight into the pathways through which MGO causes cellular damage in intestinal cells.

## 1 Introduction

Methylglyoxal (MGO) is the major compound belonging to “reactive carbonyl species” (RCS) responsible for “carbonyl stress.” It is endogenously produced under physiological conditions, as a by‐product of glycolysis and ketone body metabolism, but it is also formed by autooxidation of glucose and lipid peroxidation [1]. The digestive system can take up MGO from exogenous sources, which include dietary MGO, especially from ultra‐processed foods subjected to high temperatures [2, 3, 4, 5, 6, 7]. In addition to diet, the natural digestive tract conditions (pH and temperature) may offer a favorable environment for the Maillard reaction, leading to the formation of MGO [8]. Furthermore, lipid oxidation during gastrointestinal (GI) digestion could also increase the amounts of MGO [8].

Being a highly reactive molecule, it forms covalent adducts with several macromolecules and generates stable protein adducts with the amine‐functional groups of arginine and lysine side chains of cellular and plasma proteins, which are particularly susceptible to MGO glycation. This MGO‐induced glycation results in advanced glycation end‐products (AGE) formation. MGO adducts lead to changes in macromolecular stability and functions, and their formation is associated with cell dysfunction [1, 9]. In addition, MGO‐AGEs have been reported to impact cell functions by activating the receptor for AGEs (RAGE), which is expressed on a range of cells, including endothelial, immune, skeletal muscle, and GI cells [10, 11]. Moreover, MGO, being a small metabolite, can also enter the cell nucleus and react with nuclear proteins, including histones, and with nucleic acids, resulting in MGO‐nucleic acid adducts formation. Recent studies have also demonstrated that MGO may modulate gene expression, inducing epigenetic modifications [12, 13, 14].

Previous studies have shown that MGO and MGO‐AGEs are associated with the pathogenesis of numerous diseases including diabetes, obesity, cardiovascular and neurodegenerative diseases, and cancer [1, 6, 15, 16, 17, 18, 19]. In recent years, the effect of MGO, derived from diet and gut microbial metabolism on intestinal cells has also been investigated. The intestinal epithelium contributes to the maintenance of epithelial barrier function, and it is exposed to dietary and endogenous harmful stimuli, including MGO. Brighina et al. reported that dietary MGO could pass almost unaltered through the GI digestive stages and reach the colon [20]. MGO and other reactive dicarbonyls are transported across the intestinal cell layer via both active and passive transport and accumulate in cells [21] and it has been demonstrated that their plasmatic levels can increase from fasting to the postprandial state [22]. Finally, gut bacteria actively produce MGO via sugar metabolism, especially in the colon [23, 24, 25]. Several studies have shown that MGO and its adducts can induce GI inflammation and systemic symptoms of irritable bowel syndrome in in vivo and in vitro models [20, 26, 27, 28, 29, 30, 31]. Lin et al. also reported that MGO‐induced carbonyl stress could be a crucial promoter in colon cancer progression [32]. Moreover, MGO can modify the microbial community and cause a reduction in gut bacteria important for the microbiome [20, 25, 33, 34].

However, studies on the effect of MGO on intestinal barrier integrity and the possible underlying molecular mechanisms that could be responsible for its widespread effects are scarce [29, 30, 31]. Moreover, to date, studies on the effect of MGO on the modulation of epigenetic enzymes have not yet been carried out in intestinal cells. For this reason, intestinal barrier integrity, oxidative stress, and epigenetic regulatory enzymes that govern chromatin remodeling, DNA methylation, and histone modifications such as histone deacetylases (HDAC), ten‐eleven translocation (TET) family enzymes, and DNA methyltransferases (DNMT) were studied on an intestinal cell model exposed to MGO.

## 2 Material and Methods

### 2.1 Reagents

Cell culture reagents were obtained from Euroclone (Euroclone S.p.A., Pero, MI, Italy) and Corning (Fisher Scientific Italia, Segrate, MI, Italy). All reagents, unless otherwise stated, were purchased from Merck (Merck KGaA, Darmstadt, Germany). The fluorescent chloromethyl derivative of 2′,7′‐dichlorodihydrofluorescein diacetate (CM‐H_2_DCFDA) and Guava ViaCount solution were from Invitrogen (Invitrogen, Carlsbad, CA, USA) and Cytek (Cytek, Fremont, CA, USA), respectively.

### 2.2 Cell Culture

Human colon epithelial cells, Caco‐2 (ATCC HTB‐37), were purchased from the American Type Culture Collection (Rockville, MD, USA). Caco‐2 cells (passages 10–28) were cultured in Dulbecco's minimal essential medium (DMEM) supplemented with 1% glutamine, 1% penicillin/streptomycin, 1% essential amino acids, and 10% (v/v) fetal bovine serum (FBS) and were refreshed every 2 days before sub‐culturing when cells reached 80% confluence by trypsinization. Cells were usually seeded at a density of 5 × 10^5^ cells/well in 12‐well plates unless otherwise stated and left to grow for 24 h before MGO treatment.

For differentiation, cells were seeded in trans‐well plates (12 mm with 0.4 μm pore polycarbonate membrane insert, Corning, Glendale, AZ, USA) and differentiated for 21 days in DMEM growth medium. The medium was replaced every 2 days at both the apical (AP) and basal (BL) sides of the trans‐well filters. The integrity of the cell monolayer was checked by measuring the transepithelial electrical resistance (TEER) before and after treatment with MGO using an epithelial volt/ohm meter equipped with a chopstick electrode (Millicell ERS‐2, EMD Millipore, Billerica, MA, USA) as previously described [35].

### 2.3 Caco‐2 Cell Treatment With MGO

After reaching the right confluence, Caco‐2 cells were treated in the absence (control cells) or in the presence of MGO (MGO cells). A preliminary experiment was performed with different MGO concentrations in DMEM (0.5, 1.5, 3, 5, 10, 20 mM) and for different times (2, 6, and 24 h), and cell viability was determined using the MTT assay. A MGO concentration‐ and time‐dependent decreasing trend in cell viability was observed. Cell viability decreased significantly from the concentration of 3 mM upwards after 2 h of incubation (Figures S1 and S2). Based on these results, 3 mM MGO with a 2‐h incubation was selected as the acute exposure condition, as it allowed for the observation of molecular events. These conditions were therefore used in subsequent experiments to investigate the molecular mechanisms underlying MGO‐induced cytotoxicity and its impact on intestinal barrier integrity.

In some experiments, cell treatment with MGO was carried out in the presence of a polyphenol‐rich extract obtained from tepals of *Crocus sativus* (TE) as previously described [36]. Caco‐2 cells were incubated with 3 mM MGO in the presence of TE (100 μg GAE/mL) for 2 h. TE concentration was chosen based on a preliminary dose–response curve which showed that 100 μg GAE/mL TE was not cytotoxic and was the lowest concentration that had a significant protective effect on cell viability in MGO‐treated cells (Figure S3).

### 2.4 Cell Viability and Cytosolic ROS Detection

Intracellular ROS levels and cell viability were detected by flow cytometry as previously described [37, 38]. Briefly, after MGO treatment, cells were washed with PBS and a 1 μM CM‐H_2_DCFDA solution in DMEM (with 1% FBS) was added to each sample and incubated in the dark for 15 min at 37°C. After trypsinization, cells were harvested and centrifuged at 600 *g* for 5 min and the resulting cell pellet was resuspended in approximately 50 μL of culture medium. An aliquot of 20 μL from each sample was then added to 180 μL Guava ViaCount solution. This counterstaining was used to detect viable, apoptotic, and dead cells. The analyses for cell viability and intracellular ROS production were conducted simultaneously on a Guava EasyCyte flow cytometer (Luminex, Austin, TX, USA) using an excitation wavelength of 488 nm. Emissions were recorded using the green channel for CM‐H_2_DCFDA and the red and yellow channels for the Via‐count dye, using the following gain settings: FSC 29.3; SSC 23.6; G18.2; Y49.4; R22.6 and a threshold of 1000 on FSC. The fluorescence intensity was recorded on an average of 5000 cells from each sample. Oxidation of the ROS‐sensitive probe results in a large shift in green fluorescence, proportional to cytosolic ROS formation. For analyzing the production of cytosolic ROS, one region or gate relative to cells with high levels of green fluorescence (% of cells with high ROS) was arbitrarily set around 10% in control cells. This setting was then maintained for all experiments. The results were analyzed using In‐cyte software (Luminex, Austin, TX, USA).

### 2.5 Mitochondrial Superoxide Anion Assay

For monitoring mitochondrial superoxide anion generation, the FlowCellect MitoStress Kit (Merck Millipore) was used, which allows measurement of superoxide anion with the membrane‐permeant dye, MitoSOX Red [38]. The cell staining protocol was followed as reported in the kit's manual for cultured adherent cells. In particular, 10 × 10^5^ cells/sample were used and incubated with 5 mM MitoSOX in DMEM (with 1% FBS) for 15 min at 37°C in the dark. The analyses were conducted on the Guava EasyCyte flow cytometer using an excitation wavelength of 488 nm. Emissions were recorded using the red channel for MitoSOX Red with the following gain settings: FSC 20.7; SSC 9.93; YEL 19.9; and a threshold of 1000 on FSC. The fluorescence intensity was recorded on an average of 5000 cells from each sample, and the results are expressed as the percentage of cells with mitochondrial high ROS. Fluorescence images of the cells with the ROS‐sensitive probes were observed and captured on a Lionheart Automated Microscope (Agilent, Santa Clara, CA, USA).

### 2.6 Analysis of mRNA Expression (qPCR)

At the end of MGO treatment, total RNA was isolated using RNAzol RT (Sigma‐Aldrich, Milano, Italy). RNA concentration was determined using a nanophotometer (Implen GmbH, Munich, Germany), and its quality was determined by running all samples in 1% agarose gel stained with Xpert Green DNA Staining dye (Grisp, Porto, Portugal). DNase (MBI Fermentas, Milan, Italy) treatment was performed on all samples following the manufacturer's instructions, followed by a nanophotometer quantification of samples. Total RNA (1 μg) was then used for cDNA synthesis using the iScript cDNA Synthesis Kit (Bio‐Rad, Milan, Italy) according to the manufacturer's instructions and stored at −20°C until processed. cDNA samples were diluted 1:10 to perform qRT‐PCRs with 100 ng of template per well in duplicate using SYBR green (Bio‐Rad, Milan, Italy) in a CFX thermal cycler (Bio‐Rad, Milan, Italy) as previously described [39, 40]. The thermal profile for all reactions was 3 min at 95°C, followed by 45 cycles of 20 s at 95°C, 20 s at the primer *T*_m_ (°C) indicated in Table 1, and 20 s at 72°C. All the primers were used at a final concentration of 10 pmol/mL. Primer sequences and accession numbers are listed in Table 1. Beta‐actin (*ACTB*) and glyceraldehyde‐3‐phosphate dehydrogenase (*GAPDH*) were used as internal standards to standardize the results, eliminating variation in mRNA and cDNA quantity. Changes in gene expression levels between experimental groups are reported in the graphs as relative mRNA abundance (arbitrary units, AU).

**TABLE 1:** Primer sequences for housekeeping genes and genes of interest for qPCR.

Gene abbreviation	Gene name	Forward (5′–3′)	Reverse (3′–5′)	Accession number	Melting temperature (°C)
*GAPDH*	Glyceraldehyde‐3‐phosphate dehydrogenase	GTGAAGGTCGGAGTCAACG	GGTGAAGACGGCCAGTGGACTC	NM_002046.7	60
*ACTB*	Actin beta	ATTGGCAATGAGCGGTTC	GGATGCCACAGGACTCCAT	NM_001101.5	60
*SOD1*	Superoxide dismutase 1	GGTCCTCACTTTAATCCTCTATCCAG	CCAACATGCCTCTCTTCATCC	NM_000454.5	60
*CAT*	Catalase	TCCGGGATCTTTTTAACGCCATTG	TCGAGCACGGTAGGGACAGTTCAC	NM_001752.4	60
*HDAC1*	Histone deacetylase 1	AACCTGCCTATGCTGATGCT	CAGGCAATTCGTTTATCAGA	NM_004964.3	58
*HDAC2*	Histone deacetylase 2	GGGAATACTTTCCTGGCACA	ACGGATTGTGTAGCCACCTC	NM_001527.4	58
*HDAC8*	Histone deacetylase 8	TTTTCCCAGGAACAGGTGA	AGCTCCCAGCTGTAAGACC	NM_018486.3	54
*DNMT1*	DNA methyltransferase 1	CTACCAGGAGAAGGACAGG	CTCACAGACGCCACATCG	NM_001130823.3	58
*DNMT3A*	DNA methyltransferase 3 alpha	TATGAACAGGCCGTTGGCATC	AAGAGGTGGCGGATGACTGG	NM_006892.4	58
*hTET 1*	Ten‐eleven translocation (TET) methylcytosine dioxygenase 1	CAG AAC CTA AAC CAC CCG TG	TCG TTC GTA GCG CCA TTG TA	NM_030625.2	60
*hTET 2*	Ten‐eleven translocation (TET) methylcytosine dioxygenase 2	GAT AGA ACC AAC CAT GTT GA	TGG AGC TTT GTA GCC AGA GG	NM_001127208.3	60
*hTET 3*	Ten‐eleven translocation (TET) methylcytosine dioxygenase 3	TCC AGC AAC TCC TAG AAC TG	AGG CCG CTT GAA TAC TGA CT	NM_144993.1	60

### 2.7 Western Immunoblotting

Total cell lysates for Western immunoblotting were obtained using RIPA buffer (1% Triton X‐100, 50 mM Tris–HCl, 150 mM NaCl, 0.1% SDS, 0.5% sodium deoxycholate) containing a cocktail of protease and phosphatase inhibitors (Roche, Switzerland). Proteins were then quantified using the BCA protein assay. Proteins were resuspended in Laemmli buffer, denatured at 90°C for 5 min, and loaded (20 μg) onto 12% sodium dodecyl sulfate polyacrylamide gels for separation following SDS‐PAGE. Proteins were then transferred to polyvinylidene fluoride (PVDF) membranes overnight at 4°C for Western blotting analysis. Following blocking and washing, the membranes were incubated with specific primary antibodies for immunoblotting analysis.

The antibodies used were as follows: rabbit monoclonal Vinculin (1:1000) (A2752, Abclonal, Woburn, MA, USA), polyclonal rabbit antibody catalase (1:500) (ab16731, Abcam, Cambridge, UK), polyclonal rabbit antibody SOD1 (1:500) (ab13498, Abcam, Cambridge, UK), rabbit monoclonal Histone H2AX (1:500) (ab124781, Abcam, Cambridge, UK) and rabbit monoclonal H2AX phospho S139 (1:500) (AB81299, Abcam, Cambridge, UK), mouse monoclonal nuclear factor kappa B (NF‐κB) p65 (1:500) (sc‐8008, Santa Cruz, TX, USA), rabbit monoclonal Phospho‐NF‐κB p65 (Ser536) (1:500) (3033, Cell Signaling Technology, Danvers, MA, USA), rabbit monoclonal tumor necrosis factor (TNF)‐α (1:500) (GTX110520, GeneTex Inc., North America, USA), Histone H4 (1:500) (sc‐25260, Santa Cruz, TX, USA), and its acetylated form (1:500) (sc‐377520, Santa Cruz, TX, USA). Goat anti‐mouse IgG‐HRP (1:5000) (sc‐2005, Santa Cruz, TX, USA) and goat anti‐rabbit IgG‐HRP (1:8000) (12‐348, Merck KGaA, Darmstadt, Germany) were used as secondary antibodies. Protein bands were developed using Clarity Max Western ECL Substrate (Bio‐Rad Laboratories, Hercules, CA, USA). The chemiluminescent signal was acquired using ChemiDoc XRS + System (Bio‐Rad Laboratories, Hercules, CA, USA) and protein levels were analyzed using Image J software (Version 1.50i, National Institute of Health, Bethesda, MD, USA). The experiments were performed in triplicate and the data are presented as mean value ± SD.

### 2.8 Enzymatic Activity Assessment of TET, HDAC, and DNMT in Caco‐2 Nuclear Extracts

The ability of MGO to directly affect the activity of TET proteins, HDACs, and DNMTs enzymes was evaluated by the Epigenase 5mC Hydroxylase TET Activity/Inhibition Assay Kit (P‐3086, Epigentek, Farmingdale, NY, USA), Epigenase HDAC Activity/Inhibition Direct Assay Kit (P‐4034, Epigentek), or EpiQuik DNMT Activity/Inhibition ELISA Easy Kit (P‐3139, Epigentek), respectively [41, 42]. The nuclear extract was obtained from untreated Caco‐2 cells by utilizing the nuclear extraction kit (OP‐0002, Epigentek), according to the manufacturer's instructions. Briefly, the pellet was resuspended with NE1 buffer and centrifuged for 10 min at 12,000 rpm. Then, two volumes of NE2 buffer were added to the pellet and centrifuged at 14,000 rpm for 10 min. The Bradford protein assay was used to determine the protein concentration of the nuclear extract. Fresh extracted nuclear proteins (5 μL) were used per well for the measurement of TET (P‐3086, Epigentek), HDAC (P‐4034, Epigentek), and DNMT (P‐3139, Epigentek) activity, according to the manufacturer's instructions. Different concentrations of MGO were tested (0.001–1 mM). The absorbance was measured at 450 nm using a microplate reader, and the enzymatic activity was then calculated with the following formula:

$$\text{enzyme activity}\;\left(\mathrm{OD}/h/\mathrm{mg}\right)=\frac{\left(\text{sample}\;\mathrm{OD}-\text{blank}\;\mathrm{OD}\right)\times 1000}{\text{Protein amount}\;\left(\mu \text{g}\right)\times \text{incubation time}\;\left(h\right)}.$$

### 2.9 Statistical Analysis

All experiments were performed at least three times in duplicate and conducted in different experimental sessions. The data are shown as the mean ± SD. One‐way analysis of variance (ANOVA) was carried out using GraphPad Prism 8.2 software to evaluate any statistical differences among more than two different samples. A value of *p* < 0.05 was considered statistically significant (Tukey's post hoc multiple‐comparison test).

## 3 Results

### 3.1 Effect of MGO on Intestinal Barrier Integrity

**FIGURE 1:**
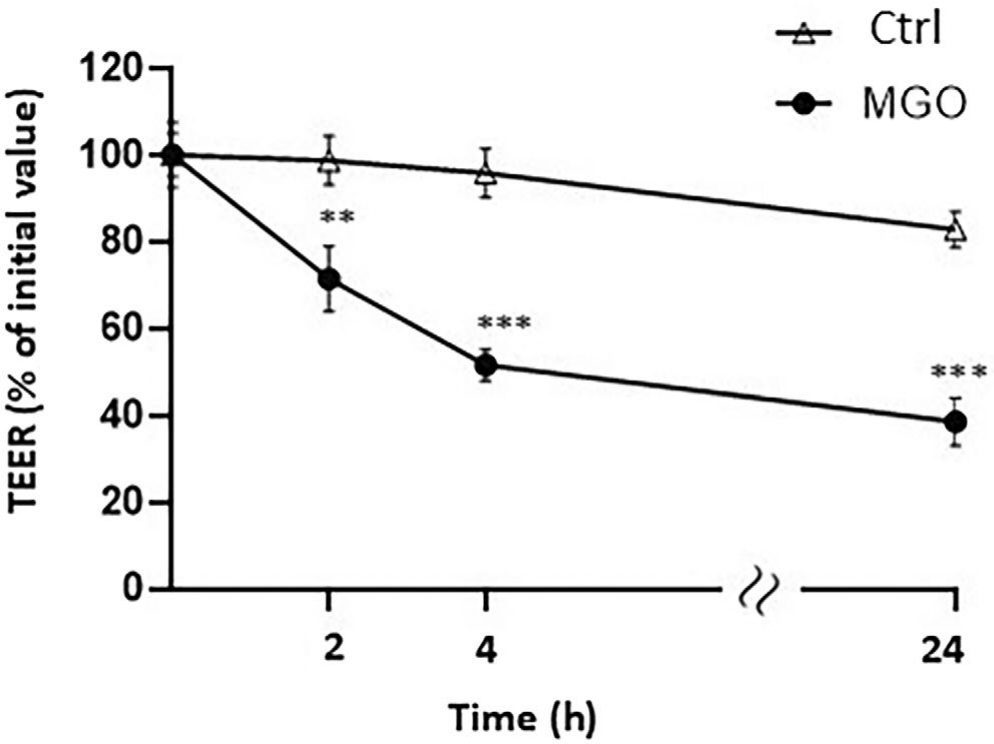
Effect of MGO on Caco‐2 monolayer cells barrier integrity. Variation in transepithelial electrical resistance (TEER) obtained by monitoring TEER in differentiated Caco‐2 cells in the absence (Ctrl) or presence of 3 mM MGO. TEER values are reported in terms of percentage with respect to the initial value. Values are presented as the mean ± SD of three determinations carried out in triplicate. Error bars represent ±SD. **p < 0.001, ***p < 0.0001 vs. Ctrl at each time point.

The evaluation of TEER across the monolayer of Caco‐2 differentiated cells was used to assess the effect of MGO on the intestinal barrier integrity. As shown in Figure 1, MGO treatment caused a significant decrease in TEER across the Caco‐2 cell monolayer compared to the non‐treated ones. After 2 h of incubation with MGO, the TEER values were 74% ± 6% of the initial value, and after 24 h of incubation, they reached 40% ± 5% of the initial value.

### 3.2 Effect of MGO on Cell Viability and Oxidative Stress

**FIGURE 2:**
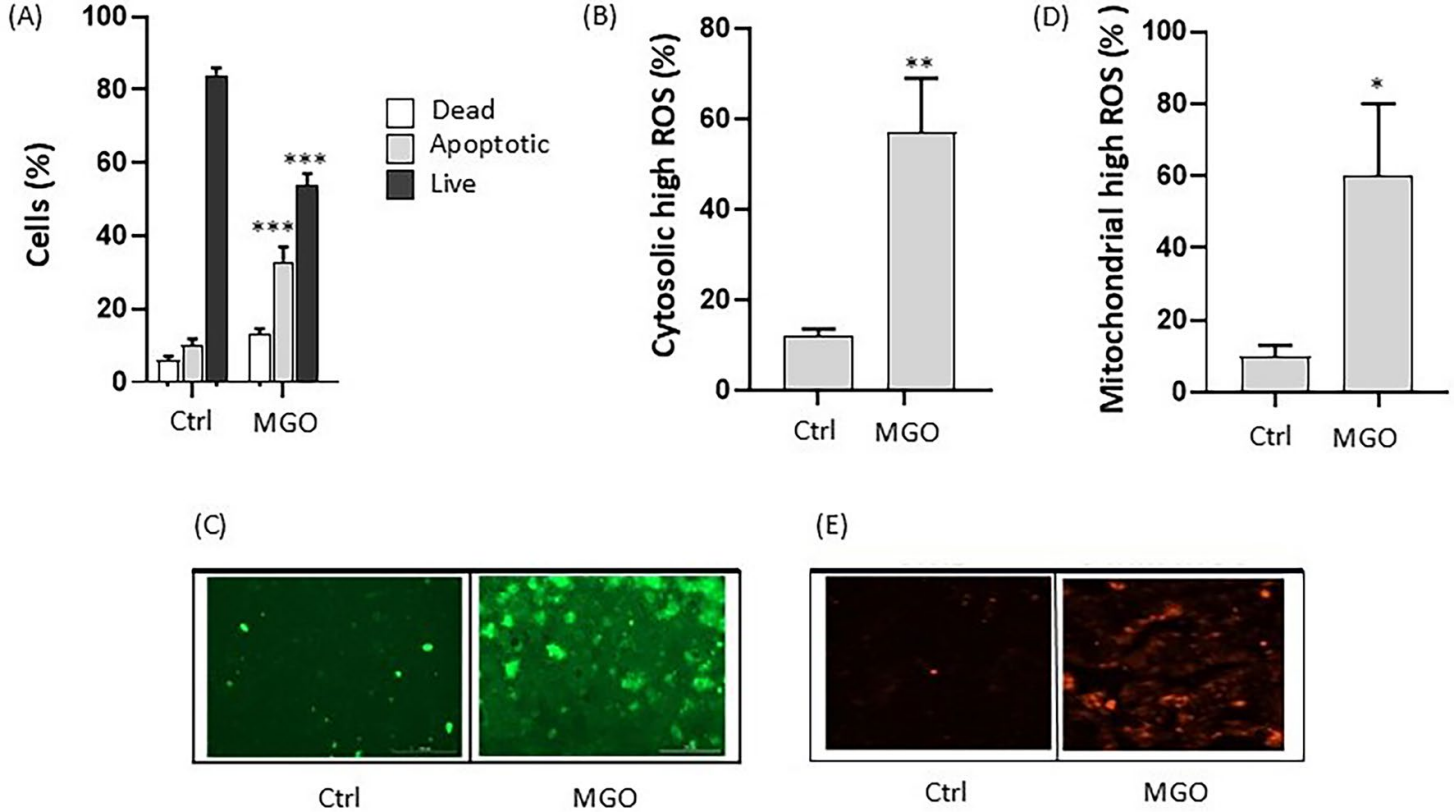
Effect of MGO on cell viability and oxidative stress in Caco‐2 cells. Flow cytometric analysis of cell viability (A), cytosolic high ROS (B), and mitochondrial high ROS (D), in Caco‐2 cells treated for 2 h in the absence (Ctrl) or presence of 3 mM MGO. Data are reported as percentage of live, apoptotic, or dead cells determined using Guava ViaCount reagent (A); as percentage of cells presenting high cytosolic ROS levels determined using flow cytometry (B) and representative fluorescence microscopy images (C) with the ROS‐sensitive probe, CM‐H_2_DCFDA; as percentage of cells presenting high mitochondrial ROS levels determined using flow cytometry (D) and representative fluorescence microscopy images (E) with the probe, MitoSOX Red. Error bars represent ±SD. *p < 0.05, **p < 0.001, ***p < 0.0001 vs. Ctrl, respectively.

The cytotoxicity of MGO was verified, as shown in Figure 2A, by flow cytometric analysis of cell viability, which demonstrated that 3 mM MGO induced a significant decrease in viable cells along with a concomitant increase in apoptotic and dead cells compared to cells incubated alone (Ctrl) after 2 h incubation.

To explore the mechanisms underlying MGO toxicity in intestinal cells, markers of oxidative stress and inflammation were investigated in Caco‐2 cells treated with 3 mM MGO for 2 h. Since MGO is a highly reactive dicarbonyl compound that is known to trigger oxidative stress, we examined intracellular ROS formation induced by MGO using CM‐H_2_DCFDA, a probe sensitive to a wide range of ROS such as hydrogen peroxide, peroxynitrite anion, superoxide radical anion, and hydroxyl radical. As shown in Figure 2B, a significant increase in the percentage of cells with high ROS was observed in MGO‐exposed cells (57% ± 12%), compared to control ones (12% ± 2%). Representative images of the fluorescence intensity indicative of ROS formation in MGO‐treated cells are shown in Figure 2C. Mitochondrial superoxide production evaluated using MitoSOX Red was also significantly elevated in MGO‐treated cells compared to control cells (Figure 2D and Figure 2E). The relative cytograms for the data reported in Figure 2 are shown in Figure S4. These data suggest that oxidative stress partakes in MGO‐induced cytotoxicity in intestinal cells.

**FIGURE 3:**
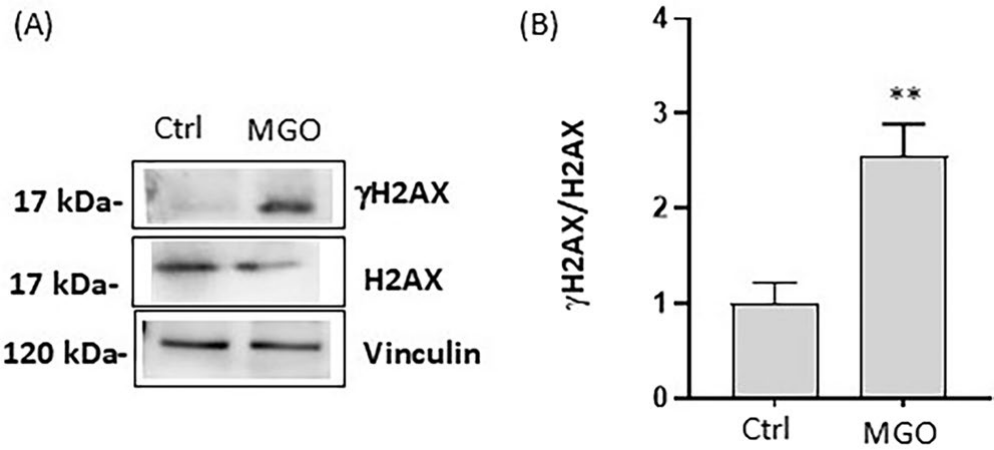
The effect of MGO on protein levels of the DNA damage marker γH2AX, in Caco‐2 cells. Caco‐2 cells were treated for 2 h in the absence (Ctrl) or presence of 3 mM MGO. (A) Representative Western blot images of γH2AX, H2AX protein expression levels and (B) corresponding densitometric analysis of their ratio normalized to Vinculin. Error bars represent ±SD. **p < 0.001 vs. Ctrl.

To further investigate the effects of MGO on Caco‐2 cells, we studied the levels of the enzyme H2AX and its phosphorylated derivative at Ser139 (γH2AX). As shown in Figure 3A, MGO treatment induced the phosphorylation of H2AX with a significant increase in the γH2AX/H2AX ratio. The ratio in MGO‐treated cells (2.55 ± 0.33) was about 2.5 times higher compared to the control ones (1.03 ± 0.22), suggesting that MGO treatment is associated with DNA damage.

**FIGURE 4:**
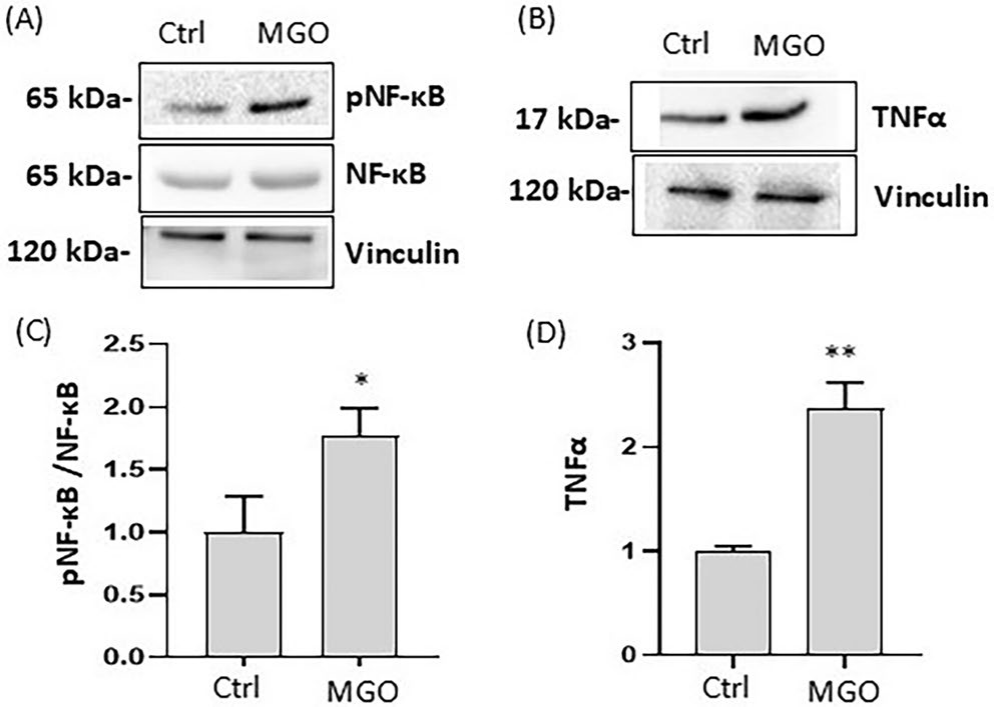
Effect of MGO on protein levels of inflammatory markers in Caco‐2 cells. Caco‐2 cells were treated for 2 h in the absence (Ctrl) or presence of 3 mM MGO. Representative Western blot images of pNF‐κB, NF‐κB, TNF‐α protein expression levels (A and B) and corresponding densitometric analysis of pNF‐κB/NF‐κB ratio and TNF‐α normalized to Vinculin (C and D). Error bars represent ±SD. *p < 0.05, **p < 0.001 vs. Ctrl.

**FIGURE 5:**
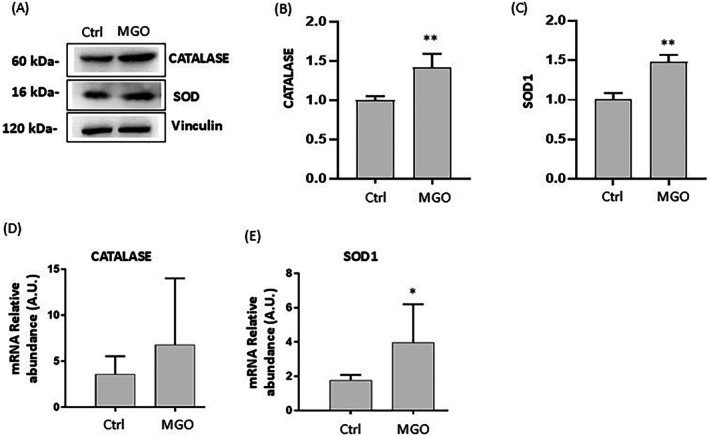
Effect of MGO on gene and protein expression of antioxidant enzymes in Caco‐2 cells. Caco‐2 cells were treated for 2 h in the absence (Ctrl) or presence of 3 mM MGO. Representative Western blot images of catalase and SOD1 protein expression (A) and their corresponding densitometric analysis normalized to Vinculin (B and C); mRNA levels of catalase and SOD1 (D and E) detected by qPCR. Error bars represent ±SD. *p < 0.05, **p < 0.001 vs. Ctrl.

To better understand the molecular mechanisms underlying MGO‐induced cell damage, we also investigated the impact on phosphorylation of NF‐κB. In MGO‐treated cells, an increase in pNF‐κB/NF‐κB ratio (1.77 ± 0.21) was observed compared to control cells (1.02 ± 0.29) (Figure 4A). These data indicate activation of this transcription factor in response to MGO‐triggered oxidative stress. NF‐κB induces the expression of various pro‐inflammatory genes, including those encoding cytokines (such as IL‐1, IL‐6, IL‐12, TNF‐α) and chemokines involved in various inflammatory processes [43, 44]. Under our experimental conditions, a significant increase in TNF‐α levels in MGO‐treated cells was observed (Figure 4B). In addition, both mRNA and protein levels of the antioxidant enzymes SOD1 and catalase were higher in MGO‐treated cells compared to control (Figure 5).

### 3.3 Evidence of Oxidative Stress Underlying MGO Cytotoxicity

**FIGURE 6:**
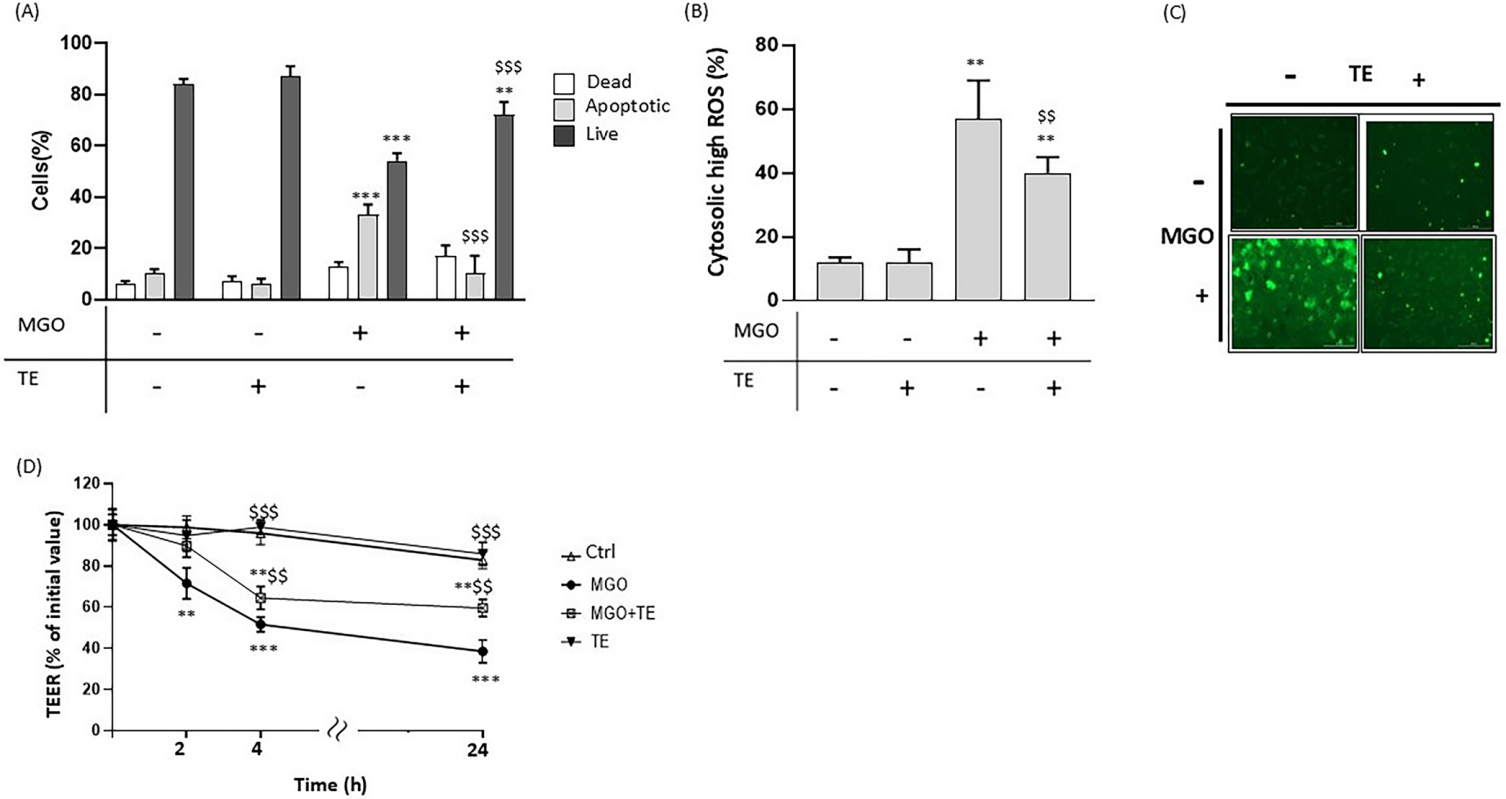
Effect of a polyphenol‐rich extract from tepals of *Crocus sativus* (TE) on cell viability (A), oxidative stress (B), and cell permeability (D) in MGO‐treated Caco‐2 cells. Caco‐2 cells were treated for 2 h in the absence (Ctrl) or presence of 3 mM MGO with or without TE (100 μg GAE/mL). Data are reported as percentage of live, apoptotic, or dead cells determined using Guava ViaCount reagent (A); as percentage of cells presenting high cytosolic ROS levels determined using flow cytometry (B) and representative fluorescence microscopy images (C) with the ROS‐sensitive probe, CM‐H_2_DCFDA; variation in cell permeability obtained by monitoring TEER in differentiated Caco‐2 cells. TEER values are reported in terms of percentage with respect to the initial value (D). Results are represented as mean ± SD of triplicate data from independent replications. Error bars represent ±SD. **p < 0.001, ***p < 0.0001 vs. Ctrl at each time point; ^$$^ *p* < 0.001, ^$$$^ *p* < 0.0001 vs. cells treated with MGO in the absence of TE at each time point.

To confirm that oxidative stress is an important mechanism by which MGO promotes cytotoxicity in Caco‐2 cells, we investigated the possible protective effect of polyphenol‐rich extracts obtained from TE. TE polyphenolic composition has been characterized, and strong antioxidant activity has been reported in our previous studies [36, 45]. For this purpose, Caco‐2 cells were treated with MGO in the presence of TE (polyphenol content: 100 μg GAE/mL). As shown in Figure 6A on cell viability, there was a significantly lower percentage of apoptotic cells and a significantly higher percentage of viable cells after treatment with MGO in the presence of TE compared to only MGO‐treated ones. A significant decrease in cells with high ROS content was observed in MGO‐treated cells in the presence of TE compared to only MGO‐treated ones (Figure 6B). No significant modification in cell viability and ROS levels was observed in cells treated with TE alone compared to Ctrl (Figure 6A,B). These results confirm that TE can counteract MGO‐induced ROS production in Caco‐2 cells. The relative cytograms for the data reported in Figure 6 are shown in Figure S5. To further confirm these data, we investigated the effect of TE against MGO‐induced intestinal barrier dysfunction in differentiated Caco‐2 cells. As summarized in Figure 6D, the presence of TE (100 μg GAE/mL) significantly increased the TEER value in MGO‐treated cells.

### 3.4 Epigenetic Modulation by MGO: Effects on DNMT, TET, and HDAC Enzyme Activity and Gene Expression in Caco‐2 Cells

**FIGURE 7:**
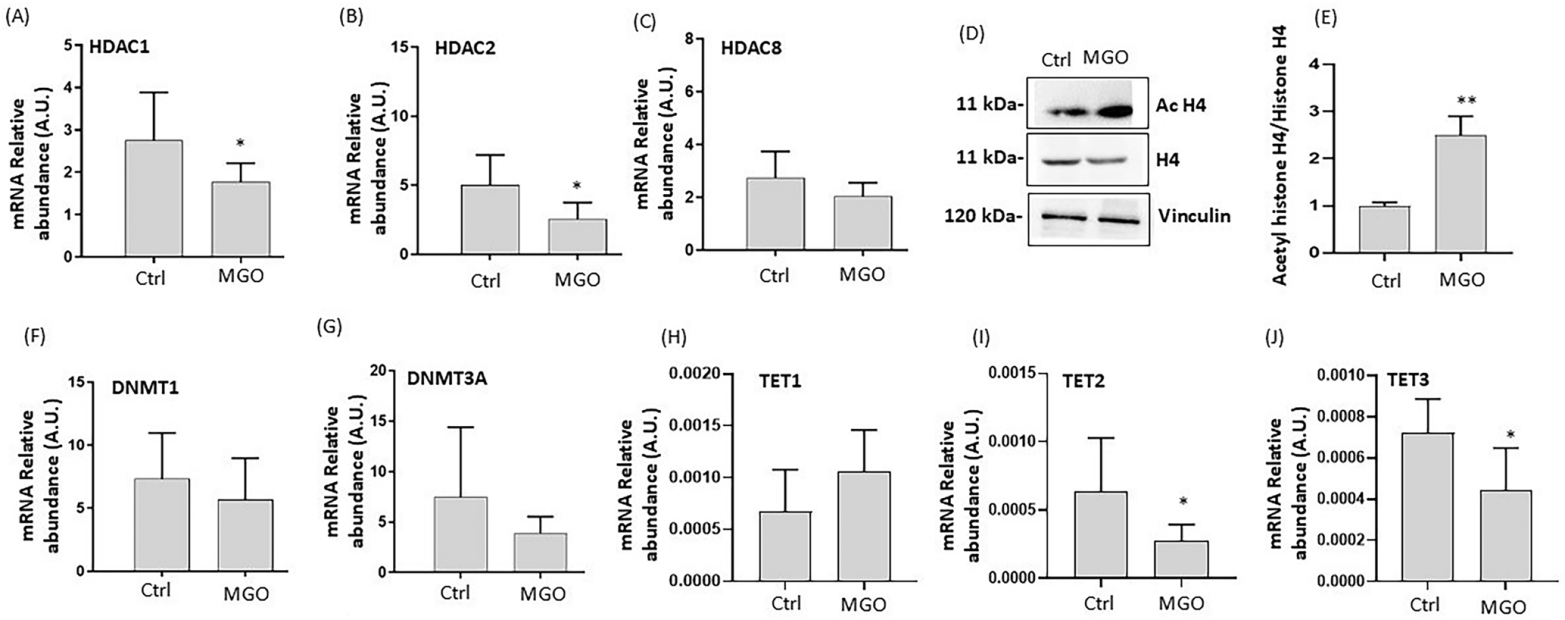
Effect of MGO on HDACs, DNMTs, TETs gene expression, and protein expression of acetylated histone H4 in Caco‐2 cells. Caco‐2 cells were treated for 2 h in the absence (Ctrl) or presence of 3 mM MGO. mRNA levels of HDAC1 (A), HDAC2 (B), HDAC8 (C), DNMT1 (F), DNMT3A (G), TET1 (H), TET2 (I), TET3 (J); representative Western blot images of histone (H4) and acetyl‐histone H4 (Ac H4) (D) and the corresponding densitometric analysis of their ratio normalized to Vinculin (E). Results are represented as mean ± SD of triplicate data from independent replications. Error bars represent ±SD. *p < 0.05, **p < 0.001 vs. Ctrl.

In order to test if the effects of MGO in intestinal cells can be attributed also to its impact on epigenetic homeostasis, we examined the expression of genes encoding enzymes involved in epigenetic regulation such as HDAC, DNMT, and TET. The results show a decrease in mRNA levels of histone deacetylases (HDAC1, HDAC2) in MGO‐treated cells (Figure 7A,B); these results are consistent with the increase in acetylated histone H4 protein levels observed in our experimental conditions (Figure 7D,E). A decrease in mRNA expression levels of DNA methyltransferases (DNMT1, DNMT3A) after MGO treatment was also observed, although not reaching statistical significance (Figure 7F,G). Of note, gene expressions of TET‐2 and TET‐3 were significantly downregulated (Figure 7I,J). In contrast, TET‐1 expression did not differ significantly between treated and untreated control cells (Figure 7H).

**FIGURE 8:**
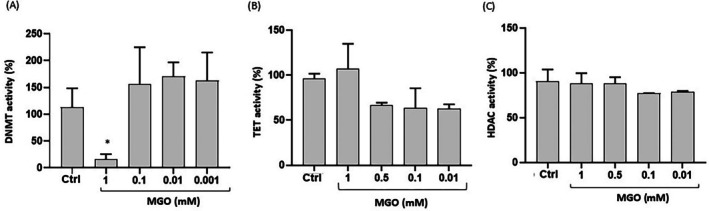
Effect of MGO on the activity of epigenetic enzymes in Caco‐2 cells. Relative activities of DNMTs (A), TET (B), and HDAC (C) enzymes in Caco‐2 cells treated with different concentrations of MGO (1–0.001 mM). Error bars represent ±SD. *p < 0.05 vs. untreated control (Ctrl).

To investigate the possible direct effect of MGO on the activity of epigenetic regulatory enzymes, nuclear extracts obtained from Caco‐2 cells were incubated with increasing concentrations of MGO. The results show that, at a concentration of 1 mM, MGO exerted a significant inhibitory effect on DNMT activity. Conversely, no significant reduction in DNMT activity was detected at lower MGO concentrations (Figure 8A). The activities of TET and HDAC enzymes remained unaffected across all MGO concentrations tested (Figure 8B,C).

## 4 Discussion

Diets abundant in ultra‐processed foods are a rich source of MGO whose formation leads to the overproduction of AGEs [2, 3, 4, 5, 6, 7]. Furthermore, diets may also contribute to MGO formation from carbohydrate metabolism by intestinal bacteria [24, 25, 26]. Several studies have now shown that the accumulation of these dietary AGEs, including MGO, is associated with organ dysfunctions, contributing to the pathogenesis of diabetes and vascular complications [15, 16, 17], thus leading to possible detrimental health outcomes [3, 6]. This occurs as a consequence of AGEs and MGO reaching the systemic circulation via the human GI tract after ingestion; hence, the intestinal barrier can act as a checkpoint for dietary AGE‐induced systemic effects [20, 21, 22].

The results from the present study show that exposure of differentiated Caco‐2 monolayers to MGO reduces the TEER value, indicating damage to intestinal barrier function induced by MGO. These data are supported by previous studies in vivo reporting that exposure to high dietary AGEs significantly increases colon permeability in rats by reducing the expression of the tight junction proteins Occludin and ZO‐1 [33]. Lim et al. have also reported that MGO‐derived hydroimidazolone‐1 (MG‐H1) induces intestinal integrity dysfunction in vitro and in vivo [29] and recently, Li et al. have shown that food‐borne dicarbonyl precursors of AGEs, including MGO, permeate the intestinal barrier, providing evidence of potential systemic bioavailability and exposure to the overall exposome, although no mechanisms underpinning this effect were investigated [21].

Our results also confirm that oxidative stress is involved in MGO‐induced intestinal dysfunction, in accordance with previous evidence [46, 47]. MGO induces excessive generation of ROS at cytosolic and mitochondrial levels, and MGO‐induced damage also involves nuclear proteins, as evidenced by the increased expression of γH2AX and the increased ratio γH2AX/H2AX. In fact, the increased ratio is considered a marker of DNA damage [48]. The involvement of MGO‐induced cellular damage was partially reversed in the presence of a polyphenol‐rich extract obtained from tepals of *C. sativus* , characterized by high antioxidant properties [36, 45]. Indeed, the presence of TE significantly decreased the generation of intracellular ROS, reduced the number of apoptotic cells, and restored intestinal barrier integrity in Caco‐2 cells treated with MGO, confirming the role of oxidative stress in MGO‐induced cellular damage. Moreover, Caco‐2 cells treated with MGO lead to the activation of NF‐κB, with further generation of ROS and overexpression of pro‐inflammatory mediators such as TNF‐α. TNF‐α is a well‐known intestinal integrity‐disrupting agent that plays a critical role in the progress of intestinal damage [49]. In accordance with our results, other studies have also reported that MGO induces activation of NF‐κB in other cell models [50, 51]. We also observed that MGO treatment increases the expression of endogenous antioxidant enzymes such as SOD1 and CAT at both mRNA and protein levels in Caco‐2 cells. This upregulation may represent a mechanism to compensate for the observed oxidative stress, thus minimizing the damaging effects of ROS induced by MGO [43].

More recent studies have demonstrated that MGO may modulate gene expression, inducing epigenetic modifications [12, 13, 14]. Therefore, we investigated the effects of MGO treatment on regulatory enzymes that govern chromatin remodeling, DNA methylation, and histone modifications such as HDAC, TET, and DNMT in Caco‐2 cells. Our results demonstrate a significant downregulation in HDAC expression levels (specifically HDAC1 and HDAC2) after MGO exposure, consistent with increased levels of acetylated histone H4, suggesting a potential boosting activity of MGO on histone acetylation processes. Literature on the role of HDACs in inflammation is fragmented, with reports on HDACs that can both inhibit and promote inflammatory responses via a multifactorial mechanism [52, 53]. Some findings suggest that increased chromatin accessibility mediated by the promotion of histone acetylation is associated with pro‐inflammatory processes [54, 55]. HDACs can alter transcriptional gene activity via chromatin‐bound histone deacetylation, thereby compacting the chromatin structure and inhibiting pro‐inflammatory gene expression. Thus, it has been proposed that HDACs can modulate the intensity and duration of inflammatory responses by regulating key signaling pathways and gene expression. For example, proper HDAC functionality inhibits TNF‐α or IL‐6 gene transcription, resulting in anti‐inflammatory effects [53]. Also, suppression of HDAC activity has been associated with NF‐κB activation [56]. Remarkably, increased acetylation of H4 was measured in both an animal model of inflammatory bowel disease and in biopsies of patients with Crohn's disease [57]. Our data also show a significant downregulation in the gene expression of TET2 and TET3 in Caco‐2 cells exposed to MGO, corroborating the hypothesis of a potential impact of MGO on DNA methylation homeostasis. These alterations are likely related to higher oxidative stress. In fact, previous studies have shown that TET proteins are affected by oxidative stress [58] and represent a potential link between redox balance and epigenetic homeostasis [59]. Indeed, TET2 and TET3 proteins have a central role in DNA demethylation processes, promoting CpG demethylation through a series of oxidative reactions [60]. Also, a recent study showed that TET2 and TET3 loss is associated with disruption of small intestine differentiation and homeostasis [61]. Our findings indicate that MGO can selectively inhibit DNMT activity, suggesting a direct impact of MGO on DNA methylation pathways. Although we did not directly evaluate DNA methylation in this study, the observed downregulation of TET2 and TET3, along with selective inhibition of DNMT activity at 1 mM MGO, may indicate a disruption of DNA methylation homeostasis. Further studies are needed to clarify whether these changes lead to measurable alterations in global or site‐specific methylation, and how they influence gene expression in intestinal epithelial cells. In contrast, TET and HDAC enzyme activities remained unaffected across all tested concentrations. Considering these findings, MGO appears to promote inflammation also by altering HDAC expression and activity in our cellular model. Since we did not measure any direct effect of MGO on HDAC activity, we can speculate that the impact on HDAC expression and acetylated H4 is mediated by the activation of indirect pathways, potentially linked to the redox system imbalance. In accordance with this hypothesis, the reduction in HDAC2 has been previously reported to be secondary to oxidative stress [62, 63] and to cause Nrf2 instability, resulting in increased sensitivity to oxidative stress [64].

Overall, although it is not currently possible to predict specific epigenomic outcomes based on our data, the simultaneous downregulation of HDAC1/2 and TET2/3 suggests an unbalanced response to epigenetic stress. On the one hand, reduced HDAC expression may lead to chromatin relaxation and transcriptional activation; on the other, decreased TET expression could impair active DNA demethylation, potentially maintaining gene silencing [65, 66]. This apparent contradiction may reflect a disorganized or nonspecific epigenetic response triggered by oxidative stress [67, 68]. Moreover, since HDAC and TET enzymatic activities were not significantly directly altered by MGO, and only DNMT inhibition was observed, our findings should be interpreted with caution and considered preliminary. Nonetheless, they support the hypothesis that MGO exposure may potentially disturb epigenetic homeostasis in intestinal cells, and that such epigenetic deregulation could constitute an additional mechanism, alongside redox imbalance, through which MGO impairs cellular health. While this hypothesis warrants further investigation, these preliminary data could pave the way for exploring specific epigenetic alterations induced by MGO in this and other cellular models.

**FIGURE 9:**
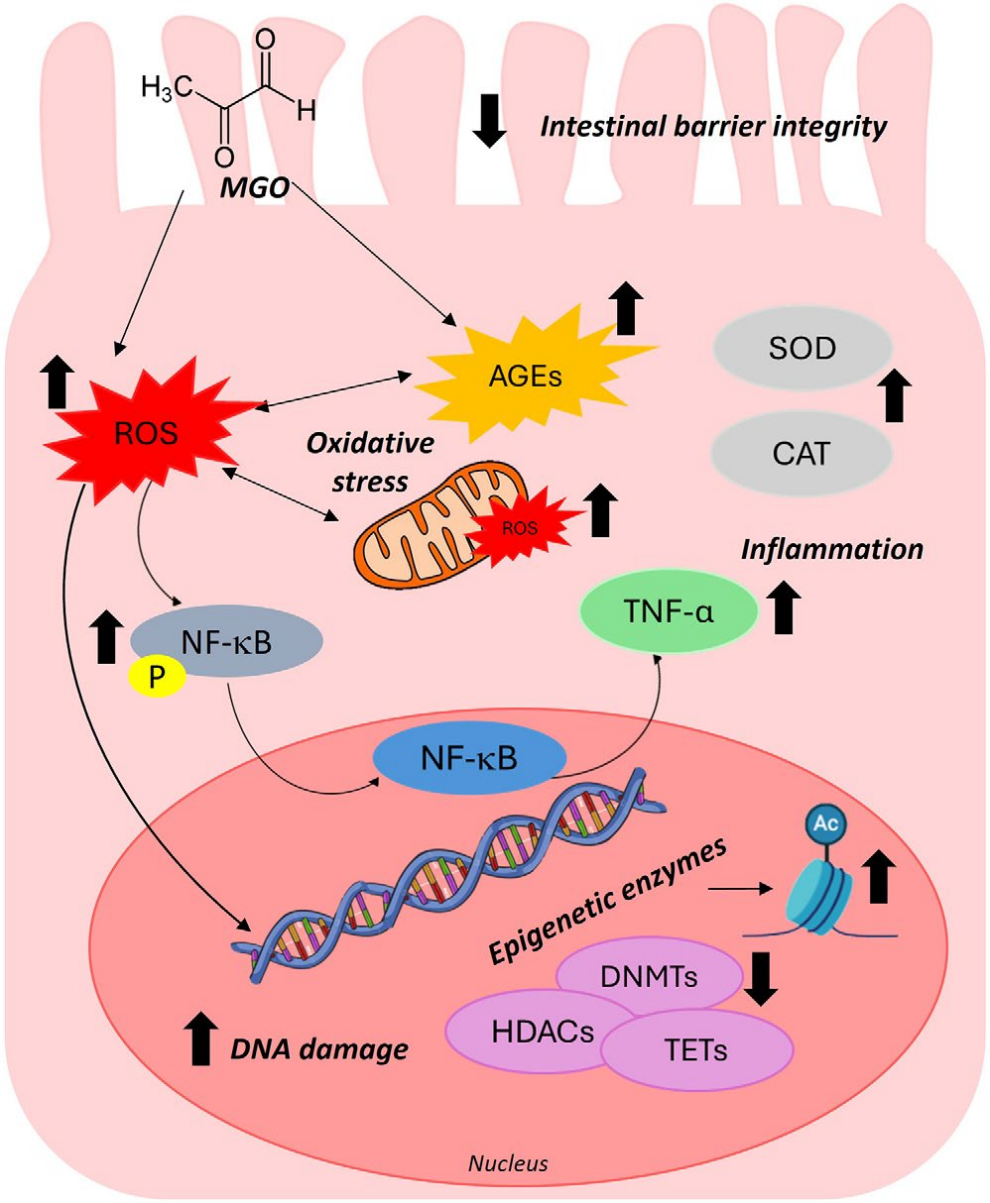
Possible mechanisms of MGO‐induced damage in intestinal cells. MGO induces glyco‐oxidative stress associated with increase in intracellular and mitochondria ROS. MGO causes inflammation by regulating the NF‐кB signaling pathway. Moreover, MGO induces DNA damage and modifications of levels of epigenetic enzymes (DNMTs, TET, and HDAC). The detrimental effects of MGO are associated with alterations integrity and functionality of the intestinal barrier.

In summary, intestinal epithelial cells represent an important defense for harmful compounds such as toxins derived from diet or bacteria, and it is continuously exposed to AGEs, including MGO. Even though MGO may be partially detoxified by glyoxalase and may react with proteins and enzymes along the GI tract prior to absorption, there are significant amounts of MGO and its derivatives that may be able to interact and be transported passively across intestinal cells, as recently reported [20, 21, 22]. Our data, summarized in Figure 9, demonstrate that MGO exposure evokes oxidative stress and inflammation triggered by high levels of cytosolic and mitochondrial ROS, and that these could represent a major upstream mechanism underlying MGO‐induced cell damage in intestinal cells. Disruption of intestinal barrier integrity caused by MGO may facilitate the translocation of MGO, dietary AGEs, and other toxic compounds from the gut lumen into systemic circulation, thereby triggering immune responses and inflammation. These cellular alterations, as well as nuclear modifications, along with early signs of potential epigenetic dysregulation, may lead to the onset of intestinal bowel diseases promoted by MGO [20, 25, 26, 28, 32, 33, 69, 70]. Furthermore, many studies have reported that a disruptive intestinal barrier function is not only found in intestinal diseases but is also associated with insulin resistance, diabetes, and other metabolic disorders such as hepatic disorders (e.g., nonalcoholic fatty liver disease) and renal failure [71].

A limitation of this study is the high concentration of MGO employed, which may not directly reflect in vivo levels. However, the precise determination of MGO concentrations at the intestinal mucosa in vivo remains challenging, as levels likely fluctuate due to dietary intake and microbial metabolism. Noteworthy is that the concentration used here is consistent with previous studies investigating the effects of MGO under similar or longer exposure conditions on Caco‐2 cells or other cellular models [26, 72, 73, 74, 75, 76, 77]. Furthermore, while Caco‐2 cells are derived from colorectal carcinoma, they are a widely accepted and well‐characterized model for studying intestinal epithelial function and barrier integrity [78, 79]. Thus, although this model does not fully capture the chronic and complex nature of in vivo MGO exposure, it provides a valuable platform to examine acute glyco‐oxidative stress and to screen botanical and non‐botanical compounds capable of mitigating the detrimental effects of dietary MGO and related reactive dicarbonyl species. Future studies will be conducted to confirm and extend these findings using complementary models, such as non‐tumorigenic intestinal cell lines (e.g., HIEC, IEC‐6) or intestinal organoid systems, and to investigate more thoroughly the potential epigenetic implications of MGO exposure and its modulation by dietary compounds.

## Funding

This work was supported by Institutional Research Funding from Polytechnic University of Marche for T.B and E.D and from the University of Camerino for R.G. Furthermore, it was also funded by NGEU PNRR, D.M. n. 118/2023 M4C1 I.4.1.

## Conflicts of Interest

The authors declare no conflicts of interest.

## Supporting information


**Figure S1:** MGO concentration‐dependent modification of cell viability evaluated with the MTT assay in Caco‐2 cells treated for 2 h. Error bars represent ±SD. *p < 0.05, **p < 0.001, ***p < 0.0001 vs. Ctrl.

**Figure S2:** Time‐dependent modification of cell viability evaluated with the MTT assay in Caco‐2 cells treated with 3 mM MGO. Error bars represent ±SD. *p < 0.05, **p < 0.001, ***p < 0.0001 vs. Ctrl.

**Figure S3:** Effect of *Crocus sativus* tepal extract (TE) on cell viability. Cell viability was evaluated with the MTT assay in Caco‐2 cells treated with increasing concentrations of TE in the absence or presence of MGO 3 mM for 2 h. Error bars represent ±SD. *p < 0.05 vs. cells in absence of TE; ^$^ *p* < 0.05, ^$$^ *p* < 0.001 vs. cells treated with MGO in absence of TE.

**Figure S4:** Representative cytograms of data reported in Figure 2 concerning the effect of MGO on cell viability and oxidative stress in Caco‐2 cells determined using flow cytometry. Cell viability (A), high cytosolic ROS (B), and high mitochondrial ROS (C) in Caco‐2 cells treated for 2 h in the absence (Ctrl) or presence of 3 mM MGO.

**Figure S5:** Representative cytograms of data reported in Figure 6 concerning the effect of *Crocus sativus* tepal extract (TE) on cell viability and oxidative stress in Caco‐2 cells treated with MGO and determined using flow cytometry. Cell viability (A) and high cytosolic ROS (B) in Caco‐2 cells treated for 2 h in the absence (Ctrl) or presence of 3 mM MGO and TE (100 μg GAE/mL).
