# Supplementary figures and images for: Effects of Methylglyoxal on Intestinal Cells: Insights on Epigenetic Regulatory Enzymes
Source: IUBMB Life. 2025 Dec 8;77(12):e70067. doi: 10.1002/iub.70067 (PMC12683318; doi:10.1002/iub.70067)

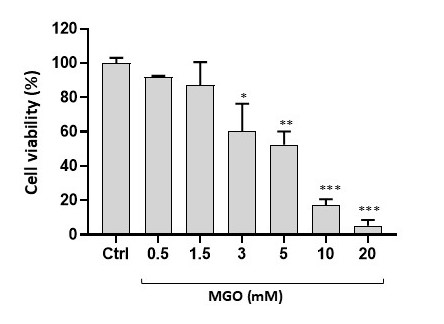

Supplement: Supplementary file 1 — Figure S1: MGO concentration‐dependent modification of cell viability evaluated with the MTT assay in Caco‐2 cells treated for 2 h. Error bars represent ±SD. *p < 0.05, **p < 0.001, ***p < 0.0001 vs. Ctrl. [file IUB-77-0-s005.jpg]

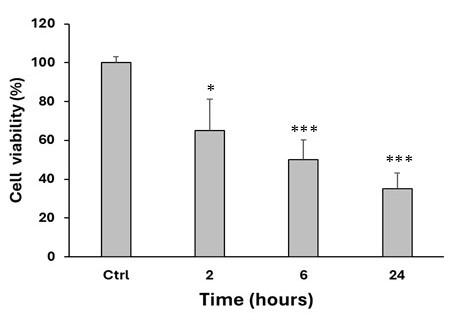

Supplement: Supplementary file 2 — Figure S2: Time‐dependent modification of cell viability evaluated with the MTT assay in Caco‐2 cells treated with 3 mM MGO. Error bars represent ±SD. *p < 0.05, **p < 0.001, ***p < 0.0001 vs. Ctrl. [file IUB-77-0-s002.jpg]

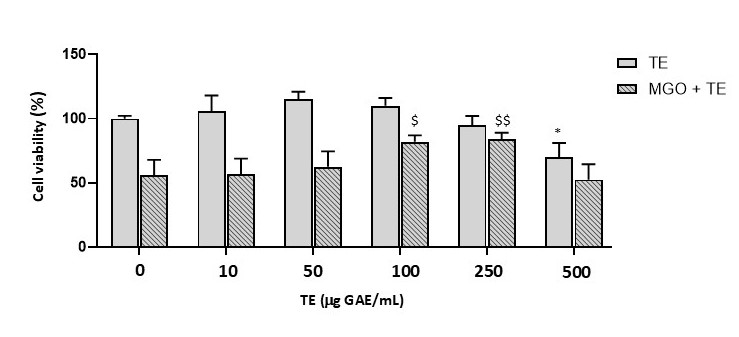

Supplement: Supplementary file 3 — Figure S3: Effect of Crocus sativus tepal extract (TE) on cell viability. Cell viability was evaluated with the MTT assay in Caco‐2 cells treated with increasing concentrations of TE in the absence or presence of MGO 3 mM for 2 h. Error bars represent ±SD. *p < 0.05 vs. cells in absence of TE; $ p < 0.05, $$ p < 0.001 vs. cells treated with MGO in absence of TE. [file IUB-77-0-s004.jpg]

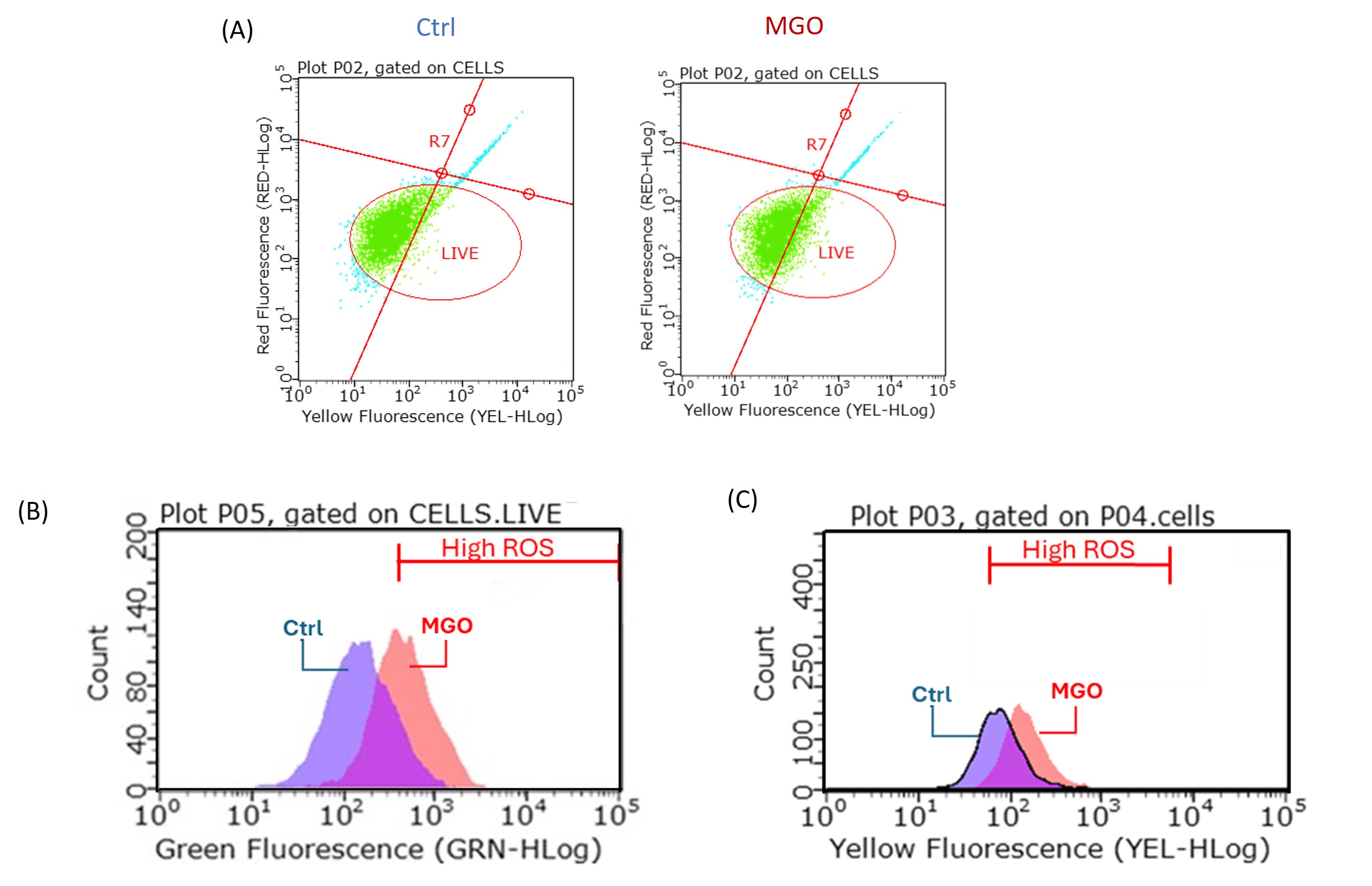

Supplement: Supplementary file 4 — Figure S4: Representative cytograms of data reported in Figure 2 concerning the effect of MGO on cell viability and oxidative stress in Caco‐2 cells determined using flow cytometry. Cell viability (A), high cytosolic ROS (B), and high mitochondrial ROS (C) in Caco‐2 cells treated for 2 h in the absence (Ctrl) or presence of 3 mM MGO. [file IUB-77-0-s003.jpg]

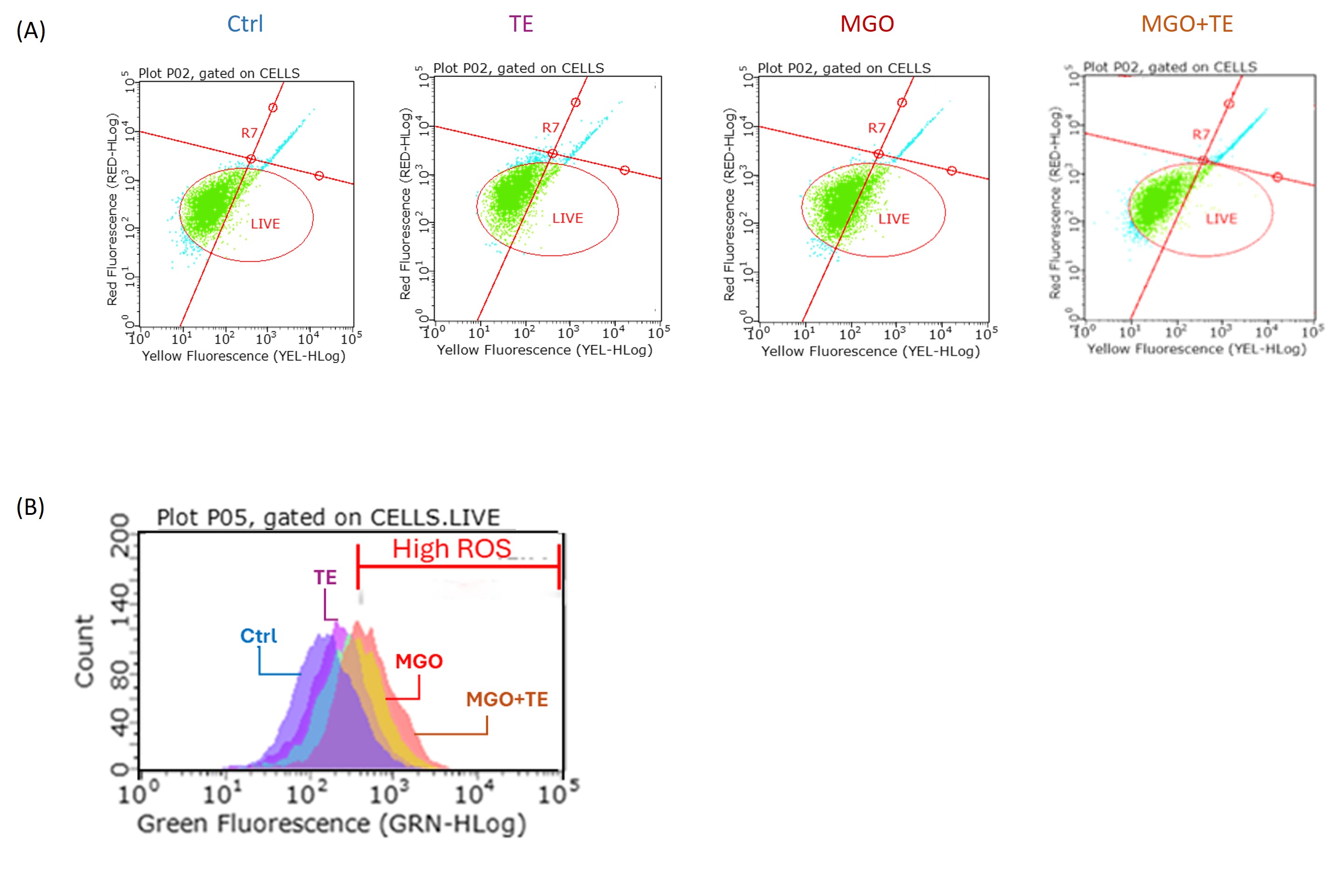

Supplement: Supplementary file 5 — Figure S5: Representative cytograms of data reported in Figure 6 concerning the effect of Crocus sativus tepal extract (TE) on cell viability and oxidative stress in Caco‐2 cells treated with MGO and determined using flow cytometry. Cell viability (A) and high cytosolic ROS (B) in Caco‐2 cells treated for 2 h in the absence (Ctrl) or presence of 3 mM MGO and TE (100 μg GAE/mL). [file IUB-77-0-s001.jpg]
